# Proteochemometric Modeling of the Susceptibility of Mutated Variants of the HIV-1 Virus to Reverse Transcriptase Inhibitors

**DOI:** 10.1371/journal.pone.0014353

**Published:** 2010-12-15

**Authors:** Muhammad Junaid, Maris Lapins, Martin Eklund, Ola Spjuth, Jarl E. S. Wikberg

**Affiliations:** Department of Pharmaceutical Pharmacology, Uppsala University, Uppsala, Sweden; Virginia Tech, United States of America

## Abstract

**Background:**

Reverse transcriptase is a major drug target in highly active antiretroviral therapy (HAART) against HIV, which typically comprises two nucleoside/nucleotide analog reverse transcriptase (RT) inhibitors (NRTIs) in combination with a non-nucleoside RT inhibitor or a protease inhibitor. Unfortunately, HIV is capable of escaping the therapy by mutating into drug-resistant variants. Computational models that correlate HIV drug susceptibilities to the virus genotype and to drug molecular properties might facilitate selection of improved combination treatment regimens.

**Methodology/Principal Findings:**

We applied our earlier developed proteochemometric modeling technology to analyze HIV mutant susceptibility to the eight clinically approved NRTIs. The data set used covered 728 virus variants genotyped for 240 sequence residues of the DNA polymerase domain of the RT; 165 of these residues contained mutations; totally the data-set covered susceptibility data for 4,495 inhibitor-RT combinations. Inhibitors and RT sequences were represented numerically by 3D-structural and physicochemical property descriptors, respectively. The two sets of descriptors and their derived cross-terms were correlated to the susceptibility data by partial least-squares projections to latent structures. The model identified more than ten frequently occurring mutations, each conferring more than two-fold loss of susceptibility for one or several NRTIs. The most deleterious mutations were K65R, Q151M, M184V/I, and T215Y/F, each of them decreasing susceptibility to most of the NRTIs. The predictive ability of the model was estimated by cross-validation and by external predictions for new HIV variants; both procedures showed very high correlation between the predicted and actual susceptibility values (*Q* ^2^ = 0.89 and *Q^2^_ext_* = 0.86). The model is available at www.hivdrc.org as a free web service for the prediction of the susceptibility to any of the clinically used NRTIs for any HIV-1 mutant variant.

**Conclusions/Significance:**

Our results give directions how to develop approaches for selection of genome-based optimum combination therapy for patients harboring mutated HIV variants.

## Introduction

The threat to human health posed by the HIV/AIDS epidemic is increasing and represents now the third largest cause of death by infectious disease in the world [1]. Since its recognition in 1981 more than 25 million people died from AIDS; only in 2008, 2 million people died, 33 million were living with HIV, and 2.7 million became infected with the virus [2]. Although the access to highly active antiretroviral therapy (HAART) has reduced the mortality in the Western world, an estimated 38,000 [estimated range 30,000–46,000] of approximately 2.25 million [1.9–2.6 million] people with HIV in the North America, Western and Central Europe died from AIDS in 2008 [2].

When given as mono-therapy, none of the available antiretrovirals is able to suppress HIV replication for any extended period of time. HAART comprises combinations of three or more drugs that aim to target HIV in different ways. First-line treatments include two nucleotide/nucleoside analog reverse transcriptase (RT) inhibitors (NRTIs) in combination with a non-nucleoside RT inhibitor (NNRTI) or a protease inhibitor [3]. RT is the sole enzyme producing double-stranded DNA from the single-stranded RNA genome, which is an essential step in the virus' replication [4]–[6]. NRTIs are analogs of nucleotide substrates that lack the 3-OH group present in the four natural deoxyribonucleotides. NRTIs are phosphorylated by cellular kinases to form 5′-triphosphates, which are used by HIV-RT as substrates and incorporated in the extending DNA chain [4], [6]. NRTIs thereby act as chain terminators, blocking further elongation of the DNA [7]. Eight NRTIs have been approved for clinical use: Zidovudine (AZT), Didanosine (ddI), Zalcitabine (ddC), Tenofovir (TDF), Lamivudine (3TC), Emtricitabine (FTC), Abacavir (ABC), and Stavudine (d4T).

Although HAART greatly increases the life expectancy of people with HIV, drug resistant virus variants emerge often. Development of resistance is primarily due to the high viral replication rate (estimated to 10^9^ to 10^10^ HIV virions per day in an average infected person [8]) and the lack of fidelity of RT with an estimated mutation rate of ∼5×10^−5^ per base per generation [9]. There are two distinct mechanisms for development of resistance to NRTIs. The first is by mutations that primarily cause steric hindrance, decreasing the rate of incorporation of the NRTI into the elongating DNA chain. The other is by mutations that increase phosphorolysis leading to removal of already incorporated chain-terminating inhibitors from the DNA, thereby allowing reverse transcription to continue.

When first-line therapy fails, the treating physician needs to select a new regimen from multiple alternative possible drug combinations. Since anti-HIV drugs acting at the same target and binding site are rather similar in their molecular properties, cross-resistance is common and a new regime cannot be based on the assumption that the virus will be susceptible to the drugs remaining in the therapeutic arsenal. Therefore, resistance testing has become an important tool in management of HIV. Such testing can be performed either by sequencing the viral genes coding for the drug targets (genotypic resistance testing), or by measuring viral activity in the presence and absence of a drug (phenotypic resistance testing). Genotypic assays are much faster and less expensive than the phenotypic ones, but sequence data provide only indirect evidence of resistance and interpretation is difficult for complex mutational combinations.

Several types of statistical and machine learning techniques have been proposed for finding relationships between mutations in the HIV genome and phenotypic drug susceptibility. These include support vector regression [10]–[12], least-squares regression [12], artificial neural networks [12], [13], decision trees [12], and non-linear regression methods [14]. However, all these models considered only one drug at a time. Hence the predictions afforded by them did not incorporate any information on similarities and differences in the structural and physicochemical properties of the antiretrovirals. This is unfortunate as it is these properties that determine the similarities and differences in a drug's interaction with drug-sensitive and drug-resistant virus variants. The models are therefore not optimal for explaining cross-resistance and they are unable to extrapolate to new anti-HIV agents. Moreover, in the previous modeling studies mutations were represented from the amino acid letter codes as binary indicator variables, rather than being based on quantitative descriptions of molecular properties of the mutated sequence residues that are relevant for drug-protein interactions (e.g. amino acid size and shape, hydrophobicity, charge, etc.). For this reason the models have limited ability to generalize and predict the effect of less common mutations on virus-drug interactions.

It is important to realize that *in vitro* susceptibility is only one of the factors to consider for drawing clinical inferences. Models have earlier also been developed to predict therapy outcome from virus genotype using clinical markers (viral load and CD4^+^ cell count), data on drug combinations in previously failed treatment regimens, and patient data (age, gender, mode of virus transmission, and adherence), as additional parameters in the modeling [15], [16]. However, these models still do not include any structural or physico-chemical data and hence cannot extrapolate to new mutations and novel drugs. Although the models show fair predictive ability (the accuracy of EuResist prediction engine being 76% when the full feature set is available for the prediction) presumably because of the inability to generalize to new mutations and drugs, these models do not include recently approved drugs for which less clinical data has been collected, such as for example the HIV protease inhibitors darunavir and tipranavir.

For quite some time we have been developing a multivariate modeling approach, termed proteochemometrics (PCM), that can perform concomitant analysis of the interactions of multiple proteins with multiple ligands. In PCM interaction activity data are correlated to the physicochemical and structural descriptions of proteins and ligands and their-derived protein-ligand cross-description, using a suited multivariate data modeling technique. In this way interpretable and predictive interaction models are created that are able to generalize to new protein-ligand combinations, and to new proteins and new ligands [17]. We have previously applied PCM for the analysis of drug interactions with different classes of G-protein coupled receptors, [18]–[24], antibody-antigen interactions [25], cleavability of protease substrates [26], and HIV resistance to protease inhibitors [27]. Here we aimed to create a generalized PCM model for predicting the susceptibility of mutated HIV variants to the clinically used NRTIs. The approach presented here to model susceptibility of antiretrovirals might find use in genome-based optimization of HIV therapy.

## Results

### Development and evaluation of the PCM model

The data set used herein comprised 728 HIV variants with unique RT sequences, covering phenotypic assays for eight NRTIs; in total the data set comprised 4,495 drug-RT combinations. As detailed in the *Methods* section, the eight NRTIs of the study were characterized by seven principal components derived from 58 molecular descriptors representing properties related to molecular geometry, flexibility, and the ability of the inhibitor to form different types of non-covalent interactions (Table S1). In the following, these seven principal components will be denoted *I* descriptor block. The 165 mutated positions in the RT sequences were each encoded by three physicochemical z-scale descriptors, totally giving 165×3 = 495 variables (*R* descriptor block). The ability for inhibitor-specific interactions of HIV mutants was described by inhibitor-RT cross-terms (*I×R* block) and eventual cooperative effects of sequence mutations were represented by cross-terms between RT descriptors (*R×R* block). Several models of varying complexity were created from these descriptions in order to find the model that provided the highest predictive ability and best interpretability.

*Model-1* included only inhibitor and RT descriptors (*I* and *R* blocks, 7+495 = 502 **X** variables); *Model-2* used inhibitor and RT descriptors together with inhibitor-RT cross-terms (*I*, *R* and *I×R* blocks, 502+7×495 = 3,967 **X** variables); *Model-3* used additionally 1,128 intra-RT cross-terms (i.e. *I*, *R*, *I×R*, and *R×R* blocks, 3,967+1,128 = 5,095 **X** variables). The logarithmically transformed change in susceptibility (log fold-decrease in susceptibility, here abbreviated log*FDS*) was used as the response (**y**) variable. Correlation of the **X** variables to **y** was performed by partial least-squares projections to latent structures (PLS).

Model performances are summarized in Table 1. *Model-1* could only partially explain the variation in inhibitor-RT susceptibility, the squared correlation coefficient (*R^2^*) being 0.57. *Model-2* performed substantially better; the *R^2^* being 0.92. These results were expected since *Model-1* used merely a linear combination of drug and RT descriptors and hence could explain only the linear part of interactions (i.e. mutations that lead to cross-resistance). Due to the cross-terms, *Model-2* can locate inhibitor-RT mutant property combinations that lead to decrease of mutated virus susceptibility to one or few of drugs, while showing lower influence or even opposite effects on other inhibitors. The large difference between the two *R^2^* values indicates a high degree of non-linearity in inhibitor-RT interactions, which results in differing resistance profiles for the different NRTIs.

**Table 1:** Performance of proteochemometric models for prediction of HIV-1 RT drug susceptibility.

Model	Descriptor blocks	Goodness of fit (*R* ^2^)	Predictive ability (*Q* ^2^)	External predictions (Q^2^_ext_)
*Model-1*	*I*, *R*	0.57	0.55	0.51
*Model-2*	*I*, *R*, *I×R*	0.92	0.87	0.85
*Model-3*	*I*, *R*, *I×R*, *R×R*	0.95	0.89	0.86

In the table I and R represent the descriptors of the inhibitors and the reverse transcriptases, respectively. I×R represents the cross-terms of descriptors of the inhibitor and the reverse transcriptase. R×R represents the intra-reverse transcriptase cross-terms (i.e. cross-terms formed from descriptors of different amino acids in the reverse transcriptase sequences).

*Model-2* was used to identify the most influential RT descriptors. Of the 495 descriptors, 48 obtained variable importance in projection (VIP) values larger than one and were used to choose intra-RT cross-terms (see Methods for explanation of VIP and for further details on selection of intra-RT cross-terms). Adding intra-RT cross-terms (*Model-3*) gave a further improvement, thus confirming the existence of cooperative effects of mutations in the transcriptase in the development of resistance.

For all three models, the predictive ability, as assessed by 7-fold cross-validation, was very close to the goodness of fit; for *Model-3* the cross-validated squared correlation coefficient *Q^2^* was as high as 0.89. However, since the results of cross-validation were used in model optimization (i.e. selecting scaling weight for cross-terms and number of extracted PLS components) there is a risk for a bias in the *Q^2^* estimate, and we wanted to ascertain that these high *Q^2^* values were not overoptimistic. To this end we performed external predictions by building the models on the data for only 70% (503 of 728) of RT sequences and setting aside the remaining 225 sequences as an independent test-set. As seen from Table 1, the external prediction results correlate well with the goodness of fit and cross-validation results for the three models; the margin between *R^2^* and *Q^2^_ext_* is 0.09 or less and the margin between *Q^2^* and *Q^2^_ext_* is as small as 0.02–0.04. The difference between the goodness of fit and the predictive ability of a PLS model can be seen as a measure for the risk of having chance-correlations with irrelevant descriptors in the model, which further on could give rise to erroneous interpretations of the physical relationships underlying the model. Alternatively the difference could result from the presence of outliers in the data-set, which would also bias the interpretations [28]. The small differences between these measures for all our three models verify that they can be used reliably for interpretations.

**Figure 1:**
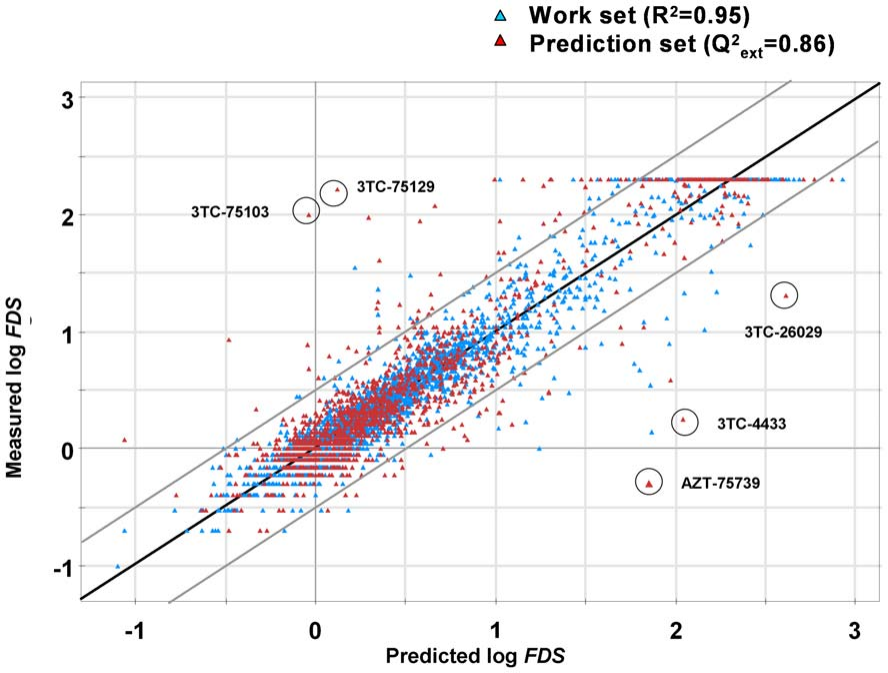
Predictive ability of the HIV RT proteochemometric model. Illustrated is the external predictive ability of the proteochemometric model (*Model-3*) for HIV drug susceptibility. The predicted versus measured susceptibility values are shown as red triangles. Goodness-of-fit of the models (i.e. model data) are shown as blue triangles.

The performance of *Model-3* is summarized in Table 2, presenting the root mean squared error of prediction (*RMSEP*) as well as the first quartile (*Q1*), median, and third quartile (*Q3*) of the absolute values of prediction errors for each of the eight inhibitors. As seen from the table, the average *RMSEP* is 0.25 logarithmic units, the best results being obtained for ddI, ddC, d4T, ABC, TDF, and FTC. The average *Q1* is 0.07, the median 0.13, and the *Q3* 0.23 logarithmic units. The performance of *Model-3* is also illustrated graphically in Figure 1. Inspection of the figure reveals that altogether about 94% of the predictions fall in the area between the oblique grey lines indicating an error of ±0.5 logarithmic units. In less than 1% of the cases (13 out of 1372) the prediction errors exceed ±1 log units (not shown graphically). A closer inspection of the data presented in Figure 1 reveals that eight of the thirteen largest mispredictions occurred for 3TC, while five occurred for AZT. A feasible explanation for this is that for these two inhibitors the *FDS* values often exceeded the upper limit of the assay, which we had approximated to *FDS* = 200 for the sake of the modeling (see Methods). Obviously this may lead to over or underestimations of the impact of mutation combinations on drug susceptibility in heavily mutated virus variants. For 3TC an additional explanation is that this drug is highly influenced by mutations of a single residue, M184, to V or I. A point mutation at this position is present in more than half the data-set sequences and brings about an, on the average, 30- to 100-fold loss of susceptibility for 3TC (*vide infra*). Inspection of the sequences of the four isolates harboring this mutation, but which retained high susceptibility to 3TC, indicates that although V (Valine) was the dominant amino acid at position 184, genotyping had detected also the wild type amino acid at this position; thus the virus isolate actually contained a mixture of several strains. Similarly, in three of the four virus isolates that were predicted falsely to be susceptible to 3TC, genotyping along with the wild type amino acid M identified also the mutant M184V. Thus, the mispredictions can be explained by the discrepancy between the results of genotype and phenotype assays; in some cases the genotypic assay identified M as the prevailing amino acid but in the phenotypic assay the dominant amino acid was actually V, while in some other cases the situation was the opposite.

**Table 2:** Prediction errors for the eight NRTIs in proteochemometric model (*Model-3*).

NRTI	Cross-validation results				External predictions			
	RMSEP*	Q1	Median	Q3	RMSEP	Q1	Median	Q3
3TC	0.34	0.07	0.16	0.26	0.37	0.07	0.14	0.24
ABC	0.17	0.04	0.09	0.17	0.20	0.05	0.12	0.20
AZT	0.42	0.11	0.21	0.40	0.47	0.12	0.24	0.48
d4T	0.14	0.04	0.08	0.15	0.18	0.04	0.09	0.16
ddC	0.16	0.05	0.10	0.17	0.17	0.05	0.09	0.17
ddI	0.12	0.03	0.07	0.14	0.15	0.04	0.08	0.15
FTC	0.21	0.06	0.14	0.20	0.25	0.05	0.12	0.23
TDF	0.23	0.05	0.12	0.19	0.22	0.14	0.12	0.24
Average	0.22	0.06	0.12	0.21	0.25	0.07	0.13	0.23

*RMSEP – root mean squared error of prediction; Q1 – first quartile; Q3 – third quartile.

Analyzing the PLS regression equation further reveals that the over-predicted *FDS* for isolate 75739 versus AZT (shown as the AZT-75739 encircled point in bottom right corner of Figure 1) arises mainly due to an F77L mutation. All virus variants in the work-set bearing this substitution are AZT resistant (log*FDS* = 1.7–2.3). Moreover, the RT of isolate 75739 contains several atypical mutations, such as T215H, which is not present in any of the work-set sequences. Since histidine is more hydrophilic than the amino acids that predominate at this sequence position (i.e. T, Y, and F), the 75739 isolate resides outside the modeled physico-chemical space. Accordingly the model derives the estimate for the isolate by extrapolation. It is also appropriate here to mention that PLS modeling provides tools for detection of outliers to identify situations when predictions must be used with caution. This includes the distance-to-model, *DModX*, criterion [29], which for the AZT-75739 pair exceeded grossly the critical value in the model; the *DModX* (normalized) was 1.83; the critical value (at the default significance level of 0.05) was 1.11.

The few discrepancies observed are not unique to our approach and have been reported previously for genotype/phenotype data [12] and even in between different phenotypic assays [30]. It is not expected that the influence of rare mutations should be possible always to be fully accounted for in statistical modeling. Still, despite the few mispredictions, the high *Q^2^* and *Q^2^_ext_* values of our model manifest its robustness and suggest that quantitative assessment can be done on the impact of RT mutations on the susceptibilities for each one of the NRTIs in the model. Although the RT sequences in the test-set harbored, on average, eleven mutations, the root mean squared error of prediction (*RMSEP*) was as low as 0.25, which indicates a good model performance also for heavily mutated virus variants.

### Analysis of the role of individual amino acids in drug resistance

Interpretation of a PLS model can be based on the analysis of the coefficients of its regression equation. E.g., a positive value for a coefficient for a z_1_-scale descriptor of an RT residue reveals that a mutation to a more hydrophilic amino acid at this position should (on the average) lead to decreased susceptibility to the NRTIs, whereas a negative value indicates the opposite. Comparisons of coefficients for all three z-scale descriptors of a mutated sequence position thus allow delineation of physicochemical and structural properties of amino acids that are responsible for the change in susceptibility. This in turn allows for predictions of the effect for mutations to any amino acid, including to such that are not present in the virus variants in the model training data-set.

**Figure 2:**
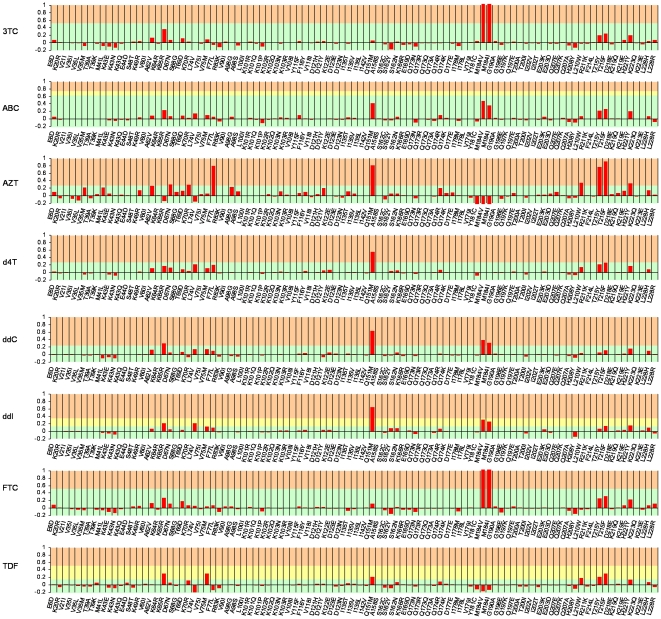
Effects of amino acid mutations on NRTI susceptibility. Illustrated are the changes in susceptibility to the eight NRTIs on single amino acid mutations in the wild type HIV-1 RT. Shown are the decimal logarithms of the fold-changes in susceptibility calculated from the proteochemometric model (*Model-3*). The green, yellow, and red areas in each panel represent susceptibilities falling, respectively, below the lower cut-off, between the lower and upper cut-off, and above the upper cut-off levels for resistance; the cut-offs being as defined by the PhenoSense assay documentation [http://www.monogramhiv.com/phenosense_report.aspx]. For some NRTIs, only one cut-off value is defined (shown in the figure by omission of the yellow zone); the cut-off for ddC (a discontinued NRTI) is set arbitrarily for illustration purposes only.

The model also takes into account of interaction effects. A large absolute value of a coefficient for a cross-term between RT and inhibitor descriptors reveals that mutations of the represented sequence residue induce large changes in the susceptibilities for some particular inhibitors and not-so-large or even opposite changes in the susceptibilities for other inhibitors. A large absolute value of a coefficient for an intra-RT cross-term pinpoints mutation pairs in the RT that regulate drug resistance in a cooperative manner. However, a detailed interpretation that includes the effects of cross-terms is difficult to describe in a written account like this one. This is due to the large number of cross-terms that needs be taken into account simultaneously. An alternative, simpler approach is to use the whole regression equation to predict drug susceptibilities of *in-silico* mutated virus variants. This is done in Figure 2, which presents the predicted changes in virus susceptibilities to the eight inhibitors due to single point mutations in the wild type RT sequence. Shown are the 99 most frequent mutations; each of them was found in over 9% of the isolates in the data-set.

The analysis of Figure 2 shows that 18 mutations reduce the susceptibility to one or several NRTIs by more than 0.2 log units. Some of these mutations afford inhibitor-specific effects. For example, mutations L210W and T215Y/F are the most deleterious for the thymidine analogues AZT and d4T, although they also reduce the susceptibility to the other NRTIs. Similarly, mutation M184V/I confers high resistance to the two structurally very similar inhibitors 3TC and FTC; it also affects susceptibility to ABC, DDC, and DDI, but is unimportant or even beneficial for AZT, d4T, and TDF. In contrast, some other mutations, such as Q151M exert similar influence on most of NRTIs (except on 3TC and FTC).

Figure 2 also discloses that quite many polymorphic mutations (i.e. reflecting natural variations in the RT) as well as some of the drug-pressure induced mutations may cause hyper susceptibility to certain inhibitors. For example, mutations M184V/I and K65R, which are the most deteriorating for 3TC and FTC, lead to significant increase in susceptibility to AZT, thus suggesting a benefit of combining these inhibitors. (However, co-formulated 3TC/AZT is only considered an alternative for first line therapy, due to adverse effects of AZT [3]).

### Online prediction of susceptibility resulting from accumulated mutations

Although some single point mutations are sufficient to render HIV resistant to individual drugs, escape from combination regimens requires accumulation of multiple mutations, which often appear in specific patterns. Cooperative effects that augment drug resistance and/or compensate for loss of virus viability are well known for many sets of mutations. E.g., a pathway that the virus uses often to escape from thymidine analogs is by the mutation T215Y (or T215F), followed by M41L, which in turn is followed by L210W [31]. As seen in the above-presented Figure 2, the model does not find that mutations of residues 41 and 210 alone are influential on the susceptibility to d4T. However, analysis of the regression equation reveals that large regression coefficients have been given to the cross-terms between the descriptors of these sequence positions and position 215, thus indicating the possibility for strong cooperative effects within the mutation triplet.

**Figure 3:**
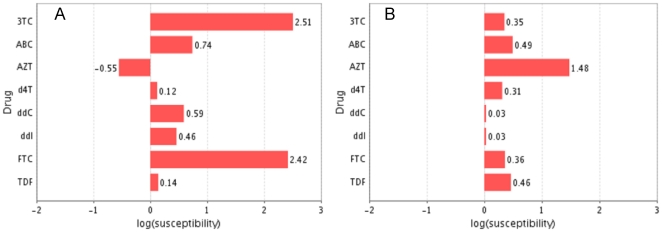
Prediction server for NRTI susceptibility. Shown are screenshots from the output from the web-page at www.hivdrc.org, utilizing the web-service based on the proteochemometric model (*Model-3*). Shown are the predicted susceptibilities for the eight NRTIs covered by the model for two sets of HIV mutant strains; K65R+M184V (panel A), and M41L+L210W+T215Y (Panel B). The web service takes an HIV-1 RT sequence as input and predicts the virus susceptibility to the NRTIs using the unified proteochemometric model.

The number of all possible combinations of mutations in the RT is immense and it is therefore beyond any practical possibility to present their complete analysis in a written report. Therefore, to facilitate predictions of the drug susceptibility of any HIV RT variant bearing any clinically known or hypothetical mutation pattern, we have set up a Web service to allow free public access to our model. The service makes use of the novel XMPP protocol [32], and a web page for invoking the service is available at [33]. Figure 3 presents screenshots of outputs from the web page, illustrating the predicted susceptibilities for two patterns of mutations: K65R+M184V (Panel A) and M41L+L210W+T215Y (Panel B). We can there see that the double mutant K65R+M184V is highly resistant to 3TC and FTC, while it shows essentially unchanged susceptibility to d4T and TDF and is hypersusceptible to AZT. On the other hand, the RT variant that harbors the triple mutation M41L+L210W+T215Y is highly resistant to AZT, while it shows an unchanged susceptibility to ddC and ddI. By comparing the susceptibility profiles in the two panels one can see that co-administration of AZT and ddI would defer each of these resistance development pathways. On the other hand, once all five mutations have accumulated in one HIV variant, this would confer resistance to all of the available NRTIs, which is a stage that poses a challenge for current anti HIV therapies.

## Discussion

In this study we applied proteochemometrics modeling to analyze susceptibilities of multiple HIV mutants to eight clinically used nucleoside/nucleotide analog reverse transcriptase inhibitors. We represented the sequences of RT variants by physicochemical property (z-scale) descriptors, rather than using letter codes for amino acids. This approach enabled us to develop models that can be used to assess the contributions of distinct physico-chemical properties of sequence residues and their combinations for the induction of resistance to RT inhibitors, and it allows us to perform predictions for new RT sequences, provided that they fall within the mutational space of polymorphic or drug-pressure created mutants of the HIV-1 subtype B.

We assessed the predictive ability of the obtained PLS model by cross-validation and by predicting a large independent test-set comprising more than 200 RT mutants. The data-sets of earlier studies addressing HIV-RT drug resistance vary both in size and diversity. It is therefore impossible to straightforwardly compare the performance of different computational techniques for modeling the drug resistance from the literature data. However, to the best of our judgment, the resolution of our PCM model, being characterized by *Q* ^2^ *_ext_* = 0.86 and *RMSEP* = 0.25, is superior compared with the resolution of the other hitherto best-performing approaches, which analyzed the susceptibilities for each NRTI in separate models [11]–[13]. This is best illustrated by comparing our results with those of the previously reported models that were also based on Stanford HIV DB data. Thus, in a study by Rhee et al. [12] regression models were created for six NRTIs exploiting least-squares regression (LSR), support vector regression (SVR), and least angle regression (LARS) and the models had been evaluated by 5-fold cross-validation. The mean values of squared correlation coefficients between the actual and predicted log*FDS* values (i.e. *Q^2^*) were for these models, respectively, 0.36, 0.53, and 0.72. The best results were achieved for 3TC (*Q^2^* being, respectively, 0.76, 0.84, and 0.93), whereas the worst predictions were for TDF (*Q^2^* being 0.01, 0.34, and 0.40) (However, it shall be noted that the study of Rhee et al. comprised 639 isolates from the Stanford database whereas our study contained 728 isolates so the results are not entirely comparable). It is notable that no models were created for ddC and FTC in the Rhee et al. study, which are inhibitors for which comparatively little amount of data have been collected in the Stanford HIV DB. While the reason for this could be that ddC was discontinued in 2006, at least for FTC it could be that insufficient data were available to create a model with the methods chosen. Again this contrasts to the PCM approach, which is able to accommodate good predictions also for a new inhibitor for which only little data is available.

In another recent study by Kjaer et al. [13], genotype-phenotype correlation models were induced for all clinically used NRTIs (except for ddC), by use of artificial neural networks (ANN). The quality of ANN models had been estimated by 10-fold cross-validation and the squared correlation coefficients between the predicted and observed log susceptibility values (i.e. *Q^2^*) ranged from 0.56 (for TDF) to 0.88 (for 3TC), with the average value being 0.74. The mean squared errors in these models ranged from 0.06 (corresponding to 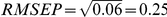) for ddI to 0.98 (corresponding to 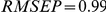) for AZT, the average being 0.26 (corresponding to 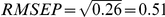). Thus, although exact comparisons of our results with the results of Kjaer et al. [13] is impossible due to the differences in the analyzed data sets, the listed *Q^2^* and *RMSEP* values points out that these models, which just covers one inhibitor at a time, are inferior to the here presented unified PCM model.

**Figure 4:**
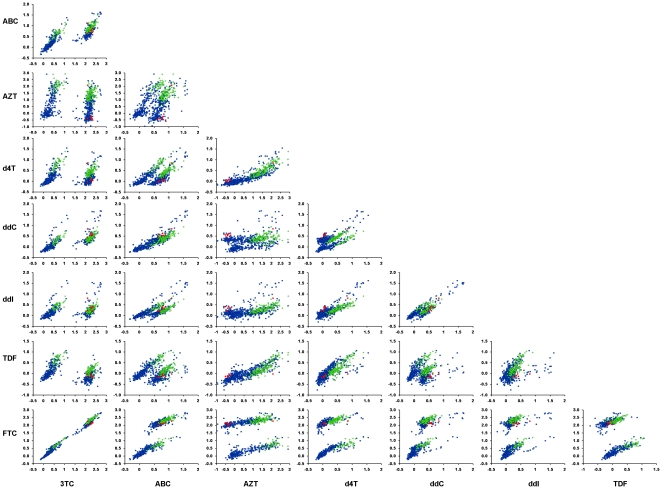
Pair-wise comparisons of NRTI susceptibilities. Shown are pair-wise comparisons of NRTI susceptibilities for mutant HIV variants, predicted from the proteochemometric model (*Model-3*). The figure represents the computed susceptibilities for all mutation combinations occurring in the data-set; red symbols represent predictions for the seven RT variants in the data-set that contained the double mutations K65R+M184V; green symbols represent the susceptibilities for 153 variants containing the triple mutations M41L+L210W+T215; the blue symbols represent predictions for 568 other RT variants in the data-set retrieved from the Stanford HIV database.

The web service developed herein has a potential to guide inhibitor selection for combination regimens against particular RT variants. Moreover, the model might also be used to derive general guidelines for inhibitor combinations for HIV variants harboring known sets of mutations. A pair-wise comparison of predicted susceptibilities for the eight NRTIs for 728 viral isolates is shown in Figure 4. For example, as can be seen from the figure, combining 3TC with TDF gives high chances that the virus is susceptible to at least one of the drugs. By contrast, co-administration of 3TC with FTC gives no benefits according to this analysis. Also, according to the analysis co-administration of ABC with FTC is the least optimal among the recommended [3] NRTI combinations for initial therapy (see Figure 4). In fact these results arise due to that a mutation of residue 184 confers resistance to both ABC and FTC. Moreover, aside from this mutation, there is also a very high correlation between the susceptibilities of the two drugs. By contrast, ABC and AZT have different resistance patterns for most mutants (Fig. 4). As shown in the figure, the analysis can be performed for any pair of drugs for the whole mutational space of HIV RT, as well for any HIV variant in a patient, and might therefore be used to find improved treatment strategies.

Thus, to sum up we have here shown how proteochemometric modeling can create a statistically valid unified model for predicting the susceptibility of HIV to NRTIs from virus genome, and we have shown how it can be used to analyze resistance patterns for combination treatments, thus giving a direction how genome-based HIV therapy can be optimized. As our modeling approach is completely general it can straightforwardly incorporate new NRTI inhibitors, and even so for new inhibitors where little data has yet accumulated for their resistance patterns. We have also made available a free web service where the model can be used to predict the resistance pattern for existing and novel mutated HIV RT variants for eight NRTIs. This web service can be found at www.hivdrc.org.

## Materials and Methods

### Data-set

Phenotypic susceptibility data estimated by the PhenoSense assay for the eight clinically approved NRTIs, Lamivudine (3TC), Abacavir (ABC), Zidovudine (AZT), Stavudine (d4T), Zalcitabine (ddC), Didanosine (ddI), Emtricitabine (FTC) and Tenofovir (TDF), were retrieved from the Stanford HIV Drug Resistance Database [34]. The PhenoSense assay measures the concentration of the anti-HIV drug required to inhibit the replication rate of an HIV isolate by 50%; i.e. the drug's IC_50_ value. The susceptibility is then expressed as the fold change in IC_50_ compared to a well-characterized drug-sensitive reference virus, the NL4-3 HIV-1 strain. The resulting quotient (IC_50_ for the tested virus divided by the reference IC_50_) is termed Fold Decrease in Susceptibility (*FDS*); a value higher than 1 indicates a decrease in susceptibility. The measurement range for the PhenoSense assay is for most NRTIs 0.1 to 200. For two NRTIs, namely AZT and 3TC, approximate *FDS* values exceeding 200 were reported in some cases. For these cases we set the values to *FDS* = 200 for sake of the PCM modeling.

The Stanford database also provides the sequences of the first 240 residues (i.e. the active site) of the RT sequences (i.e. data obtained by the genotypic assay) along with the HIV susceptibility data. The downloaded data-set comprised 728 HIV variants with unique RT sequences, covering phenotypic assays for totally 4,495 drug-RT combinations. Sequences contained from one to 28 (on the average 11) mutations, when compared to the HIV-1 subtype B consensus sequence reported at the Stanford database [35]. (The data-set used herein is available at http://www.hivdrc.org/data/NRTI_PhenoSense_DataSet.xlsx, and can be used for fair comparison of PCM with other modeling methods.)

For about 0.7% of the codons the genotyping had determined that a mixture of two or more amino acids was present in the data set. In most of these cases a mutated amino acid was found to be present together with the wild-type amino acid; e.g. for the resistance associated mutation M184V seventeen combinations VM and eight combinations MV were reported. For creating PCM models we used the description of the first-listed amino acid. This was justified as we in a preliminary study had explored a description utilizing the physico-chemical properties of all detected amino acids. However, such a more complex description did not give any improvement in the predictive ability of the resulting models over using the first listed amino acid only. Accordingly we did not use this approach in the subsequent modeling.

Some of the RT sequences contained so called ‘insertion complexes’, which consist of a mutation followed by the insertion of one or several amino acids; e.g. the insertion complex of residue 69, which reduces susceptibility to all NRTIs, was present in 10 of 728 sequences. Unfortunately, insertions were only indicated in the Stanford database, providing no data on how many and which amino acids that were inserted. Accordingly, we could not compare the physico-chemical properties of the inserted amino acid(s) and analyze their influence on the drug interactions. Since the insertions could not be described explicitly the proteochemometric model transferred their influence to the influence of the underlying mutation.

Since the Stanford database does not provide patient identifiers we do not know whether or not several sequences originate from the same patient. This may give a risk that the model performance is overestimated due to correlation of data originating from the same patient. (However, this risk is inversely proportional to the number of new mutations that have accumulated in a patient between the repeated measurements). On the other hand, using susceptibility data of virus strains evolving from the same patient may allow better assessment of the contribution of individual mutations on development of resistance, and may thus be beneficial for model performance.

### Numerical descriptions for PCM modeling

#### Description of Reverse Transcriptase

Of the 240 sequence residues of the RT recorded in the database, 75 were invariant while 165 contained two or more different amino acids in the set of the 728 RT sequences. We encoded each residue in the mutated positions by three z-scale descriptors [36]. These z-scales were obtained by principal component analysis [37] of 26 measured and calculated physicochemical and structural properties of amino acids and can be interpreted as representing hydrophobicity (z_1_), steric properties (i.e. size/shape; z_2_), and electronic properties (z_3_) [36]. In this way, all the differences in the physicochemical properties of the 728 RT sequences in the data-set were characterized by 165×3 = 495 descriptors. Prior to further use, descriptors were mean-centered.

#### Description of RT Inhibitors

The 3D structures of the active 5-triphosphate form of the organic compounds were obtained by modeling with the Corina unit of the Tsar 3.3 (Accelrys Inc.) software. Compounds were then characterized by molecular descriptors representing properties related to molecular shape, flexibility, and ability to form different types of non-covalent interactions. To this end descriptors of different classes were calculated using Dragon 2.1 software (Talete S.r.1), as follows: geometrical descriptors, charge and aromaticity indices, constitutional descriptors, counts of functional groups and atom-centered fragments, empirical descriptors, and molecular properties. Descriptors were checked for mutual correlation; when two or more descriptors were highly correlated (pairwise r^2^>0.95) only one of them was retained. In this way 58 molecular descriptors were obtained for modeling [38] (see Table S1 for a complete list of descriptors used).

Descriptors were mean centered and scaled to unit variance. Further on, to reduce the number of descriptors and to eliminate their mutual co-linearity, we performed principal component analysis (PCA) [37] on the data set, which transformed all descriptors into seven orthogonal principal components.

#### Reverse Transcriptase-Inhibitor and intra-Reverse Transcriptase cross-terms

Protein-ligand interactions depend on the complementarities of protein and ligand properties. In PCM these can be represented by protein-ligand cross-terms. We here calculated cross-terms by multiplying each of the protein descriptors with each of the principal components for the ligand descriptors, which yielded 7×495 = 3,465 inhibitor-RT cross-terms.

To account for eventual cooperative effects of mutations in the RT, we also introduced intra-RT cross-terms. However, deriving cross-terms between each and every one of the 495 RT descriptors would have resulted in a huge amount of variables that could give rise to chance correlations in the subsequent modeling and make model interpretations incomprehensible. We therefore elected to derive cross-terms only between the descriptors of sequence residues that appeared important for drug susceptibility. To this end we calculated variable importance in the projection (VIP) for RT descriptors in a preliminary model (*i.e. Model-2*, *vide infra*). A VIP value larger than one indicates that a variable has higher than average influence in the model [39]. Inspection of *Model-2* (see *Results*) showed that 48 RT descriptors obtained VIP>1. These most influential descriptors were used to calculate cross-terms, which accordingly gave 48×47/2 = 1128 intra-RT cross-terms.

Prior to use in the modeling all cross-terms were mean-centered. Since the number of cross-terms (i.e. 3,465 inhibitor-RT cross-terms and 1,128 intra-RT cross-terms) greatly exceeded the number of ordinary descriptors (i.e. 495 RT and 7 ligand descriptors), block scaling was applied onto the block composed of inhibitor-RT cross-terms (*I×R* block) and the block composed of intra-RT cross-terms (*R×R* block). The scaling weights of these two blocks of cross-terms were varied systematically starting from zero and increasing them gradually until an optimal model was obtained (i.e. the most predictive model according to the model's *Q^2^* parameter).

### Correlation by partial least-squares projections to latent structures

RT descriptors, inhibitor descriptors, and cross-terms were correlated to the susceptibility data, expressed as the logarithmically transformed *FDS* values (log*FDS*), by partial least-squares projections to latent structures (PLS). PLS finds a quantitative relationship between a matrix of independent variables **X** and one or several response variables (**y** vector or matrix) by simultaneously projecting **X** and **y** to latent structures (PLS components) [29]. The directions and magnitudes of the influence of **X** variables on the response **y** are revealed by coefficients in the regression equation derived by a PLS model. PLS modeling was performed using the orthogonal-PLS algorithm [40] as implemented in the Simca-P 11 software (Umetrics AB).

The goodness-of-fit of a PLS models is characterized by the fraction of explained variation of **y** (*R* ^2^). The predictive ability was characterized by the fraction of the predicted **y**-variation (*Q* ^2^), estimated by seven-fold cross-validation. *Q^2^* is calculated as:
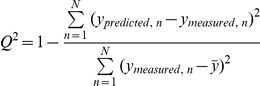
where 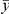
is the average of the measured outcome values for the N objects in the data-set [39]. While the goodness of fit increases by each extracted PLS component, the predictive ability typically reaches a maximum and then declines when the model becomes too complex.

Some earlier studies have indicated that a high value of *Q* ^2^ may be insufficient evidence for a model to be highly predictive, when only this parameter is used as a criterion for selecting model parameters (e.g. adjusting scaling weights for cross-terms and finding optimal number of PLS components) [41]. We therefore also performed external validation of the PLS models. For these validations the data-set was subdivided into a work-set (about 70% of the RT sequences) and a prediction-set (about 30% of the RT sequences). Splitting was performed in a random fashion by assigning to the prediction-set all sequences for which the last digit in the SeqID number in the Stanford database was 3, 6, or 9.

## Supporting Information

Table S1 List of 58 molecular descriptors used to characterize molecular properties of NRTIs for proteochemometric modeling of HIV drug susceptibility.
(0.06 MB PDF)
