# Supplementary material for: Proteochemometric Modeling of the Susceptibility of Mutated Variants of the HIV-1 Virus to Reverse Transcriptase Inhibitors
Source: PLoS One. 2010 Dec 15;5(12):e14353. doi: 10.1371/journal.pone.0014353 (PMC3002298; doi:10.1371/journal.pone.0014353)
Supplement: Table S1 — List of 58 molecular descriptors used to characterize molecular properties of NRTIs for proteochemometric modeling of HIV drug susceptibility. (0.06 MB PDF) [file pone.0014353.s001.pdf]

Table S1: List of 58 molecular descriptor types calculated by dragon 2.1 software (Talete S.r.1) used in proteochemometrics modeling of NRTI susceptibilities for HIV-1 reverese transcriptases.

Nr.	**Symbol**	**Meaning**
1	MW	Molecular weight
2	AMW	Average Molecular weight
3	Ss	Sum of Kier H all electrotroplogical states
4	Mv	Mean atomic van der Walls volume (scaled on C atom)
5	Me	Mean atomic Sanderson electronegativity (scaled on C atom)
6	Mp	Mean atomic polarizability (scaled on C atom)
7	Ms	Mean electrotopological state
8	nAT	number of atoms
9	nBO	Number of non-H bonds
10	nCIR	Number of circuits
11	RBN	Number of rotatable bonds
12	RBF	Rotatable bond fraction
13	nDB	Number of double bonds
14	nC	number of Carbon atoms
15	nN	Number of Nitrogen atoms
16	nS	Number of Sulfur atoms
17	nR05	Number of 5-membered rings
18	Qmean	Mean absolute charge (charge polarization)
19	Q^2	Total squared charge
20	RPCG	relative positive charge
21	TE2	Topographic electronic descriptor (bond restricted)
22	LDip	Local dipole index
23	MAXDN	Maximal electrotoplogical negative variation
24	MAXDP	Maximal electrotoplogical positive variation
25	TIE	E-state topological parameter
26	RGyr	Radius of gyration (Mass weighted)
27	SPAN	Span R
28	SPAM	Average Span R
29	SPH	Spherosity
30	ASP	Asphericity
31	FDI	Folding degree index
32	PJI3	3D Petijean shape index
33	G(N..N)	Sum of geometrical distances between N..N
34	G(O..O)	Sum of geometrical distances between O..O
35	nCp	Number of total primary C(sp3)
36	$nCrH_2$	Number of ring secondary C(sp3)

37	nCrHR	Number of ring tertiary C(sp3)
38	nCONHR	Number of secondary amides (aliphatic)
39	nNH_2	Number of primary amines (aliphatic)
40	nHDon	Number of donor atoms for H-bonds (with N and O)
41	nHAcc	Number of acceptor atoms for H-bonds (with N and O)
42	C-002	CH_2R_2
43	C-008	CHR_2X
44	C-024	R--CH--R
45	C-029	R--CX--X
46	C-030	X--CH--X
47	H-047	H attached to C^1(sp3) / C^O(sp2)
48	H-048	H attached to C^2(sp3) / C^1(sp2) / C^O(sp)
49	H-049	H attached to C^3(sp3) / C^2(sp2) / C^3(sp2) / C^3(sp)
50	H-052	H attached to C^O(sp3) with 1X attached to next Carbon
51	H-053	H attached to C^O(sp3) with 2X attached to next Carbon
52	N-069	Ar-NH_2 / X-NH_2
53	N-072	RCO-N< / >N-X=X
54	Ui	Unsaturation index
55	ARR	Aromatic ratio
56	MR	Ghose-Crippen molar refractivity
57	PSA	Fragment-based polar surface area
58	MLOGP	Moriguchi octanol-water partition coefficient (logP)
